# Targeted Delivery of an Antigenic Peptide to the Endoplasmic Reticulum: Application for Development of a Peptide Therapy for Ankylosing Spondylitis

**DOI:** 10.1371/journal.pone.0077451

**Published:** 2013-10-14

**Authors:** Hui-Chun Yu, Ming-Chi Lu, Chin Li, Hsien-Lu Huang, Kuang-Yung Huang, Su-Qin Liu, Ning-Sheng Lai, Hsien-Bin Huang

**Affiliations:** 1 Department of Life Science and Institute of Molecular Biology, National Chung Cheng University, Chia-Yi, Taiwan; 2 Section of Allergy, Immunology, and Rheumatology, Department of Medicine, Buddhist DaLin Tzu-Chi Hospital, Chia-Yi, Taiwan; 3 School of Medicine, Tzu-Chi University, Hualien, Taiwan; 4 Department of Nutrition and Health Science, Fooyin University, Kaohsiung, Taiwan; University of London, St George's, United Kingdom

## Abstract

The development of suitable methods to deliver peptides specifically to the endoplasmic reticulum (ER) can provide some potential therapeutic applications of such peptides. Ankylosing spondylitis (AS) is strongly associated with the expression of human leukocytic antigen-B27 (HLA-B27). HLA-B27 heavy chain (HC) has a propensity to fold slowly resulting in the accumulation of misfolded HLA-B27 HC in the ER, triggering the unfolded protein response, and forming a homodimer, (B27-HC)_2_. Natural killer cells and T-helper 17 cells are then activated, contributing to the major pathogenic potentials of AS. The HLA-B27 HC is thus an important target, and delivery of an HLA-B27-binding peptide to the ER capable of promoting HLA-B27 HC folding is a potential mechanism for AS therapy. Here, we demonstrate that a His_6_-ubiquitin-tagged Tat-derived peptide (THU) can deliver an HLA-B27-binding peptide to the ER promoting HLA-B27 HC folding. The THU-HLA-B27-binding peptide fusion protein crossed the cell membrane to the cytosol through the Tat-derived peptide. The HLA-B27-binding peptide was specifically cleaved from THU by cytosolic ubiquitin C-terminal hydrolases and subsequently transported into the ER by the transporter associated with antigen processing. This approach has potential application in the development of peptide therapy for AS.

## Introduction

Major histocompatibility complex (MHC) class I molecules present cytosolic peptides, mostly derived from proteasome-degraded fragments of intracellular proteins, to CD8^+^ cytotoxic T cells. Those peptides to be presented are transported by the transporter associated with antigen processing (TAP) from the cytosol into the lumen of the endoplasmic reticulum (ER), where they are loaded onto a heterodimer comprising an MHC class I heavy chain (HC) and a β_2_-microglobulin (β_2_m). The assembled MHC class I HC/β_2_m/peptide complex is then translocated through the trans-Golgi to the cell surface, where the peptide is presented for recognition by CD8^+^ cytotoxic T cells. 

MHC class I HC misfolding can cause human disease. Ankylosing spondylitis (AS) is an inflammatory disease characterized by inflammatory back pain and asymmetric peripheral oligoarthritis [1-4]. AS is strongly associated with the expression of human leukocytic antigen-B27 (HLA-B27) [5,6], one of the MHC class I molecules consisting of a heavy chain (α chain) and β_2_m that is assembled with a binding peptide in the ER. Several lines of evidence have indicated that the HLA-B27 heavy chain (HLA-B27 HC) has an intrinsic propensity to fold slowly in the ER before it is complexed with β_2_m and a peptide, resulting in the formation of disulfide-linked heavy-chain homodimers, (B27-HC)_2_ [7-9] and ER stress. After transport to the cell surface, (B27-HC)_2_ binds to the natural killer-cell Ig-like receptor (KIR3DL2) as well as to T-helper 17 cells (Th17), stimulating their activation and leading to the pathogenic potential of AS [10-12].

In addition, in a transgenic rat model, the ER stress induced by misfolded HLA-B27 activates the unfolded protein response (UPR), which stimulates the activation of NF-κB and promotes the expression of pro-inflammatory cytokines such as tumor-necrosis factor-α, interleukin (IL)-1, IL-6, and IL-23 [13-17]. Macrophages were activated by the UPR after accumulation of misfolded HLA-B27 HC releasing IL-23, which stimulated Th17 cells. Recent studies have indicated that the HLA-B27-binding peptide in the ER can promote HLA-B27 HC folding [18,19]. Thus, HLA-B27 HC molecules in the ER appear to be an important therapeutic target for AS. The efficient delivery of HLA-B27-binding peptides into the ER may suppress the formation of (B27-HC)_2_ and reduce the occurrence of UPR, in turn slowing down the development of AS. However, until now, no suitable method has been developed to deliver the binding peptide with accurate size and sequence to the ER.

The human immunodeficiency virus Tat-derived peptide, GRKKRRQRRR, is a small basic peptide that can translocate various types of cargo across membranes [20. 21]. After translocation, the Tat fragment is removed in a non-uniform manner in the cytosol or in the ER, resulting in cargo peptides that are mismatched with their respective MHC class I HC/β_2_m molecules in terms of length and sequence. To surmount this shortcoming, we devised a Tat-derived peptide tagged with His_6_-ubiquitin (THU) as a vehicle to deliver a cargo peptide fused to its C-terminus. The THU-cargo peptide fusion was rapidly transported into the cytosol, where the cargo peptide was released from THU by a specific cleavage reaction by cytosolic ubiquitin C-terminal hydrolases (UCHs). The liberated peptide was then transported into the lumen of the ER by TAP. In this study, two HLA-B27-binding peptides, RRFKEGGRGGKY and RRYLENGKETL, were delivered into the ER lumen using the THU vehicle. These two cargo peptides were derived from the DNA primase of *Chlamydia trachomatis* and human HLA-B27 HC, respectively [22,23] and were shown to promote HLA-B27 HC folding and decrease the formation of (B27-HC)_2_. 

## Materials and Methods

### Protein purification

The cDNAs encoding Tat-derived peptide (GRKKRRQRRR)-His_6_-ubiquitin (THU), THU-RRFKEGGRGGKY (THUC), THU-RRYLENGKETL (THUB), His_6_-ubiquitin-RRFKEGGRGGKY (HUC), and His_6_-ubiquitin-RRYLENGKETL (HUB) were generated by two-step PCR. The primers used for PCR are summarized in Table S1. The product from the first PCR served as a template for the second PCR. The product of the second PCR was subcloned into pET28a at the NheI/XhoI sites of the vector. *E.coli* BL21 (DE3) cells transformed with the recombinant vector encoding THUC, THUB, THU, HUC, or HUB were grown in 1 l of Luria broth (LB) with 0.3 g/l kanamycin sulfate at 37°C with shaking at 250 rpm. When the absorbance at 600 nm was between 0.6 and 1.0, 0.38 g isopropyl-β-D-thiogalactoside (IPTG) was added for a final concentration of 1 mM to induce recombinant protein expression. Cells were harvested by centrifugation 3 h after induction. The pelleted cells were resuspended and lysed by French Press in 30 ml of 20 mM Tris-HCl, pH 7.9, 0.5 M NaCl, 0.2 mM phenylmethanesulfonyl fluoride, 0.02% sodium azide, 4 mM benzamidine. The insoluble components were removed by centrifugation at 20,000 g for 20 min. The supernatant was loaded onto a Ni^2+^-Sepharose column (2.5 × 10 cm). After washing with one volume of the buffer used for lysis, bound proteins were eluted with a linear imidazole gradient (5 mM to 1.0 M imidazole) in the same buffer. The fractions containing the expressed protein were pooled and concentrated to 10 ml by ultrafiltration using a YM-10 membrane, and the concentrate was then diluted with 20 ml of 20 mM MOPS, pH 7.0, 0.2 mM EDTA. All components were resolved by FPLC at a flow-rate of 3.0 ml/min over a Mono-S column with a linear gradient (0 to 2 M NaCl) over 50 min. The fractions containing the target protein were pooled, dialyzed against the de-ionized water and lyophilized.

### Preparation of misfolded HLA-B-2704 HC

We constructed a vector to express a recombinant thioredoxin-truncated HLA-B27 HC (Trx-B27) fusion protein with a His_6_ tag and a thrombin cleavage site upstream of the truncated HLA-B27 HC sequence. The truncated HLA-B27 HC lacks the signal peptide and the transmembrane domain. The cDNA for the truncated HLA-B2704 HC was PCR-amplified with the following primers: forward 5′-ggctcccactccatgagg-3′ and reverse 5′-ccatctcagggtgagggg-3′. The resulting product was cleaved with BamHI/EcoRI and sub-cloned into a pET32a vector. *E. coli* BL21(DE3) cells were transformed with the recombinant pET-32a plasmid encoding the Trx-B27 fusion protein. Transformed bacteria were grown in LB with 100 μg/l ampicillin and induced with 1 mM IPTG for 4 h at 37°C. 

Most of the bacterially-expressed Trx-B27 was particulated in inclusion bodies. Insoluble Trx-B27 HC was extracted with binding buffer (20 mM Tris-HCl, pH 7.9, 0.5 M NaCl, 5 mM imidazole) containing 6 M urea. Crude extracts were purified by affinity chromatography using a Ni^2+^-Sepharose column and eluted using a linear imidazole gradient (5 mM to 250 mM) in binding buffer with 6 M urea. Eluted fractions were analyzed by sodium dodecyl sulfate polyacrylamide gel electrophoresis (SDS-PAGE). The fractions containing Trx-B27 HC were combined and dialyzed against PBS to remove excess reagents. After dialysis against PBS, the Trx-B27 HC was concentrated to 1 mg/ml by ultrafiltration. Trx-B27 HC (5 mg) was treated with 1 unit of thrombin at room temperature for 5 h. SDS-PAGE analysis indicated that the Trx-B27 HC was completely cleaved by thrombin. Cleaved Trx-B27 HC was combined with 2-mercaptoethanol (final concentration, 1%) and granular urea (final concentration, 6 M), heated in boiling water for 5 min to reduce disulfide bonds, and then subjected to gel filtration through a Superdex^TM^200 column (35 × 600 mm). Protein-containing fractions were analyzed by SDS-PAGE. The fractions containing truncated HLA-B27 HC were pooled, dialyzed against 0.2 M acetic acid, and lyophilized.

### Anti-misfolded B27-HC monoclonal antibody, BH2

A monoclonal antibody was produced by Kelowna International Scientific Inc. (Taipei, Taiwan) using misfolded HLA-B27 HC (2 mg) dissolved in 2 ml of 0.2 M acetic acid as the target antigen. The resulting anti-misfolded B27 HC monoclonal antibody, BH2, was purified by protein A-Sepharose chromatography from ascites fluids. 

### Over-expression of HLA-B2704 HC in HMy2.C1R cells

HMy2.C1R cells (ATCC, Manassas, VA) are a B-lymphoblast cells that do not express the HLA-A or -B genes. HMy2.C1R cells were maintained in Iscove's Modified Dulbecco's medium (IMDM) (Invitrogen, Carlsbad, CA) with 10% fetal bovine serum (FBS) (Invitrogen). HLA-B2704 cDNA was cloned by RT-PCR (forward primer: 5′-atgcgggtcacggcgccc-3′ and reverse primer: 5′-tcaagctgtgagagacacatc-3′) from the PBMCs of an AS patient and sub-cloned into the SalI and BamHI sites of the pTRE2hyg vector (Clontech, Mountain view, CA). The resulting vector was linearized using BamHI and transfected into HMy2.C1R cells by electroporation (Gene Pulser Xcell, BioRad Laboratories, Richmond, CA). Transfected cells were cultured in IMDM with 10% FBS and hygromycin B (200 μg/ml) (Invitrogen). After drug selection, the surviving cells expressing HLA-B27 HC were isolated and analyzed by flow cytometry. 

### Immunoprecipitation

C1R-B2704 cells were grown to confluence in a T-75 flask, harvested, and centrifuged at 300 x *g* for 5 min. After washing with PBS buffer, cells (6 x 10^6^ cells) were pelleted by centrifugation. The pelleted cells were ruptured by 0.2 ml of PBS buffer, containing 1% NP40, 1mM Na_3_ZO_4_, 1 mM phenylmethylsulfonyl fluoride, aprotinin (0.2 U/ml), and leupeptin (20 μg/ml). 400 μg of protein extract were subjected to immunoprecipitation by BH2 (20 μl) or W6/32 (20 μg) pre-immobilized on Protein G-Sepharose (50 μl). After incubation at 4°C overnight, all components were washed with the 1X NP40 lysis buffer for five times and harvested by centrifugation (12,000 x *g*) at 4°C for 1min. The immunoprecipitated molecules were analyzed by western blot using anti- BiP/Grp78 antibody (Santa Cruz Biotechnology) and BH2 monoclonal antibody.

### TAP1 knockdown

C1R-B2704 cells (1 × 10^7^ cells/ml) were transfected with 10 μg TAP1 shRNA plasmid (Santa Cruz Biotechnology, Santa Cruz, CA) following the methods described by the manufacturer. The transfected cells were cultured in IMDM with 10% FBS, 200 μg/ml hygromycin B, and 0.4 μg/ml puromycin (Invitrogen). After antibiotic selection, TAP1 protein expression was analyzed by immunoblotting with an anti-TAP1 antibody (Abcam, Cambridge, UK) and an HRP-conjugated goat anti-mouse IgG for detection by ECL (Amersham Biosciences, Piscataway, NJ). 

### Ethics Statement

 Enrollment took place between January, 2010 and December, 2012 in Buddhist Dalin Tzu-Chi General Hospital, Chia-Yi, Taiwan. All of the participants signed informed consent forms approved by the Institutional Review Board and Ethics Committee (IRBEC) of Buddhist Dalin Tzu-Chi General Hospital. The protocol for isolation of human PMBCs from AS patients described blow has been reviewed and approved by IRBEC. The IRBEC approval number is B09801021. A written informed consent document has been obtained from each participant. Human PBMCs from the AS patients were prepared as previously described [24].

### Patients

 The patients enrolled in this study were AS patients defined according to the modified New York criteria [25]. All AS patients were HLA-B27-positive and included 17 males and 3 females. The mean age of the patients was 34.4 ± 11.2 years. 

### Western blot analysis

C1R-B2704 cells (3 × 10^6^ cells/well) were seeded on 24-well plates and maintained in 1 ml IMDM with 10% FBS and 200 μg/ml hygromycin B. C1R-B2704 cells (3 × 10^6^ cells/well) that had been treated to knockdown TAP1 were maintained in the same medium with 0.4 μg/ml puromycin. Cells were treated with 20 μg of THUC, THU, HUC, THUB, or HUB and harvested at the time point indicated. Membrane proteins were extracted using a ProteoExtract Native Membrane Protein Extraction Kit (Calbiochem, Darmstadt, Germany). Freshly prepared 10 mM iodoacetamide was included in all buffers to block disulfide bridge formation during membrane protein extraction. The supernatant containing the extracted membrane proteins was collected, and aliquots of extracted membrane proteins were resolved by non-reducing SDS-PAGE (10%). The resolved proteins were transferred to polyvinyl difluoride membranes (Amersham Biosciences) and immunoblotted with BH2 monoclonal antibody followed by horseradish peroxidase (HRP)-conjugated goat anti-mouse IgG (Santa Cruz Biotechnology). The cognate molecules were visualized by enhanced chemiluminescence. 

### Flow cytometry

C1R-B2704 cells (2 × 10^6^ cells/well) or TAP1-knockdown C1R-B2704 cells (2 x 10^6^ cells/well) were seeded in 24-well plates and maintained in 1 ml IMDM with 10% FBS, 200 μg/ml hygromycin B and 0.4 μg/ml puromycin. Cells were treated with 10 μM THUC, 10 μM THUB, 10 μM THU, or 10 μM HUC, 10 μM HUB overnight, washed with PBS three times and stained with W6/32 antibody (Abcam, 1:500 dilution) in the dark for 30 min. The stained cells were washed with PBS three times, incubated with goat FITC-conjugated anti-mouse IgG (Millipore, Temecula, CA; 1:500 dilution) in the dark for 30 min, washed with PBS three times, and analyzed by flow cytometry. 

### Stimulation of PBMCs with THUC

 PBMCs isolated from HLA-B27-positive AS patients were resuspended in RPMI 1640 medium (Invitrogen) with 10% FBS, 100 units/ml penicillin, and 100 μg/ml streptomycin. PBMCs (5 × 10^6^ cells) in 1 ml medium were treated with 5 μM THUC or THUB and incubated at 37°C with 5% CO_2_. After 48 h, human recombinant interleukin 2 (*BD Biosciences*, Bedford, MA, Cat. No. 354043) was added to a final concentration of 50 IU/ml [26]. After eight days, the CD8^+^ T cells were isolated by using the Human CD8 T Lymphocyte Enrichment Set-DM kit (BD IMag^TM^, San Diego, CA, Cat. No. 557941) according to the manufacturer’s instructions.

### The CD8^+^ T lymphocyte (CTL)-mediated cytotoxicity assay

C1R-B2704 cells or TAP1-knockdown C1R-B2704 cells were used for the CTL-mediated cytotoxicity assay. One milliliter C1R-B2704 cells (1 × 10^6^ cells) or 1 ml TAP1-knockdown C1R-B2704 cells (1 × 10^6^ cells) was treated with THUC (20 μg), THUB (20 μg) or THU (20 μg) overnight. After washing with PBS three times, the targeted cells were diluted to 5 × 10^4^ cells in 100 µl RPMI 1640 medium containing 10% FBS, 100 units/ml penicillin, and 100 μg/ml streptomycin, and stimulated CTL cells (2.5 × 10^5^ cells in 100 μl) were added. All components were incubated in a 96-well plate with V-shaped bottoms for 4 h at 37°C with 5% CO_2_. Cells were washed once with 1 ml PBS, fixed in 1 ml 4% paraformaldehyde for 15 min at 4°C, and permeabilized using FACS Permeabilizing Solution 2 (BD, Cat. No. 340973) for 15 min at room temperature. The fixed and permeabilized cells were removed from the solution by centrifugation at 1200 rpm, and the cell pellet was washed with 1 ml PBS. The cells were subsequently stained with a FITC rabbit anti-active caspase-3 antibody (clone C92-605, BD Pharmingen^TM^, Cat. No. 559341) for 30 min, washed with PBS, and resuspended in 250 μl PBS for analysis by flow cytometry.

### Endoglycosidase H (Endo H) assay

 C1R-B2704 cells (3 × 10^6^ cells/well) were seeded in a 24-well plate and maintained in 1 ml IMDM with 10% FBS and 200 μg/ml hygromycin B. Cells were treated with 20 μg of THUC, THU, HUC, THUB, or HUB for 24h. The membrane proteins were extracted by the above-mentioned method. An aliquot (2 μg) of the membrane proteins was treated with one unit of Endo H (New English Biolabs, Beverly, MA; P0702S) at 37°C for 2 h. The resulting product was analyzed by reducing SDS-PAGE, western blotting and probed for B27 HC. 

### Statistical analysis

Data are presented as means ± SD. Statistical significance was assessed by unpaired Mann–Whitney *U* tests for comparing numerical data between different groups. *P*-values less than 0.05 were considered statistically significant.

## Results

To demonstrate the application of THU as a vehicle for translocation, we stably overexpressed HLA-B2704 HC in HMy2.C1R cells (C1R-B2704). The HMy2.C1R cell line is a B-lymphoblast cell line that does not express the HLA-A or -B genes [27]. HLA-B2704, an MHC class I molecule, is an HLA-B27 subtype highly associated with the pathogenesis of AS [1] that demonstrates slow folding kinetics and misfolding in the ER. 

**Figure 1:**
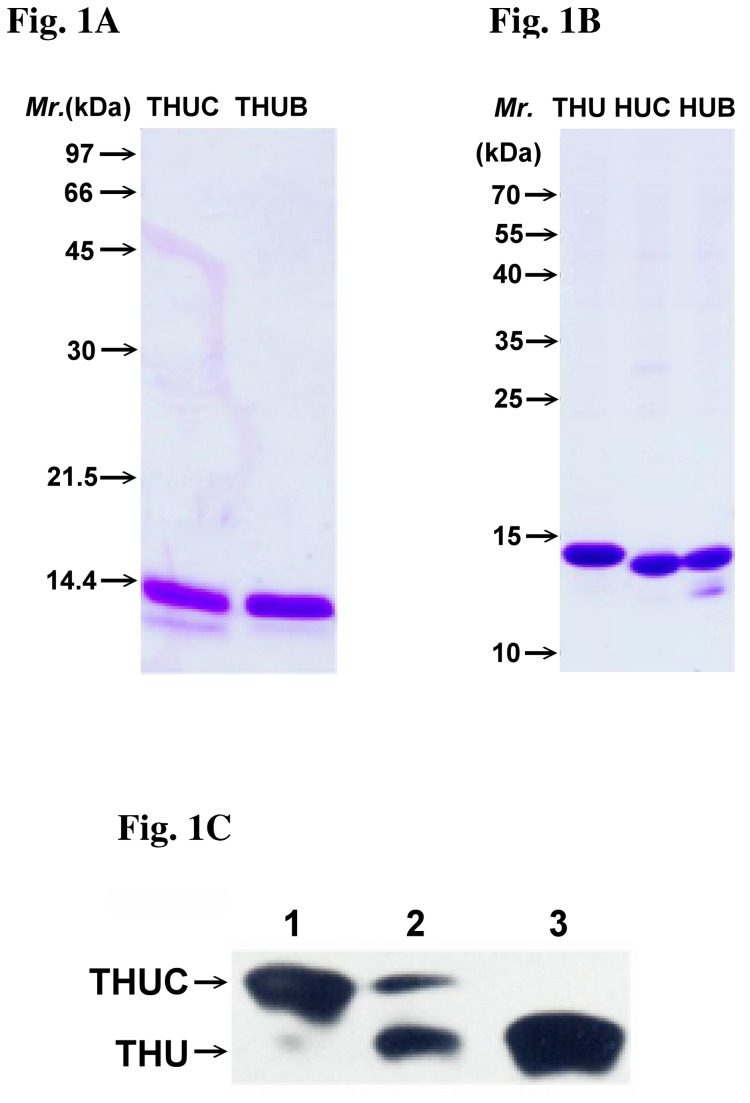
Analysis of the recombinant proteins. (A) Recombinant THUB and THUC were resolved by SDS-PAGE (15%) and stained with Coomassie Brilliant Blue. Lane 1: THUC (2 μg) ; Lane 2: THUB (2 μg). (B) Recombinant THU, HUC, and HUB were resolved by 15% SDS-PAGE and stained with Coomassie Brilliant Blue. Lane 1: THU (2 μg); Lane 2: HUC (2 μg); Lane 3: HUB (2 μg). (C) The cargo peptide is cleaved from THUC intracellularly. C1R-B2704 cells (2 × 10^6^ cells) in 1 ml IMDM were treated with 20 μg THUC for 4 h. The cells were pelleted by centrifugation. Proteins were extracted with 100 μl of 1% NP40, resolved by SDS-PAGE, and immunoblotted using an anti-His_6_ antibody. Lane 1: THUC (100 ng); Lane 2: Total protein extracts (20 μg); Lane 3: THU (100 ng).

In this study, we delivered two HLA-B27-binding peptides, RRFKEGGRGGKY and RRYLENGKETL, to the ER of C1R-B2704 cells and to peripheral blood mononuclear cells (PBMCs) isolated from AS patients. These two cargo peptides were derived from the DNA primase of *Chlamydia trachomatis* and human HLA-B27 HC, respectively [22,23]. Tat-derived peptide (GRKKRRQRRR)-His_6_-ubiquitin (THU), THU-RRFKEGGRGGKY (THUC), THU-RRYLENGKETL (THUB), His_6_-ubiquitin-RRFKEGGRGGKY (HUC), and His_6_-ubiquitin-RRYLENGKETL (HUB) were overexpressed in *E. coli* and then purified by Ni^2+^-Sepharose and cation exchange FPLC (Figure 1A and 1B). Both cargo peptides were cleaved from THUC and THUB by yeast UCH1 in an in vitro assay (Figure S1A and Figure S1B ). The results consistent with a similar cleavage by endogenous UCHs in C1R-B2704 cells were observed, as evidenced by the western blotting (Figure 1C). The cleaved peptide from THUC was analyzed by MALDI-TOF, indicating that the yeast UCH1 precisely cleaved the C-terminus of ubiquitin (Figure S2). We have prepared a recombinant protein, THUX. The X represents a peptide, HRVACISTR. THUX containing the sole Cys in the X peptide was labeled with 5-iodoacetamido-fluorescein (5-IAF). C1R-B2704 cells were treated with the AF-labeled THUX. We examined whether the cleaved AF-labeled X peptide can be translocated into the ER by fluorescence microscopy. Figure S3 indicates that the cleaved peptide is co-localized with calreticulin, a protein marker of ER, suggesting that the AF-labeled peptide can be cleaved from THUX by the cytosolic UCHs and translocated into the ER.

**Figure 2:**
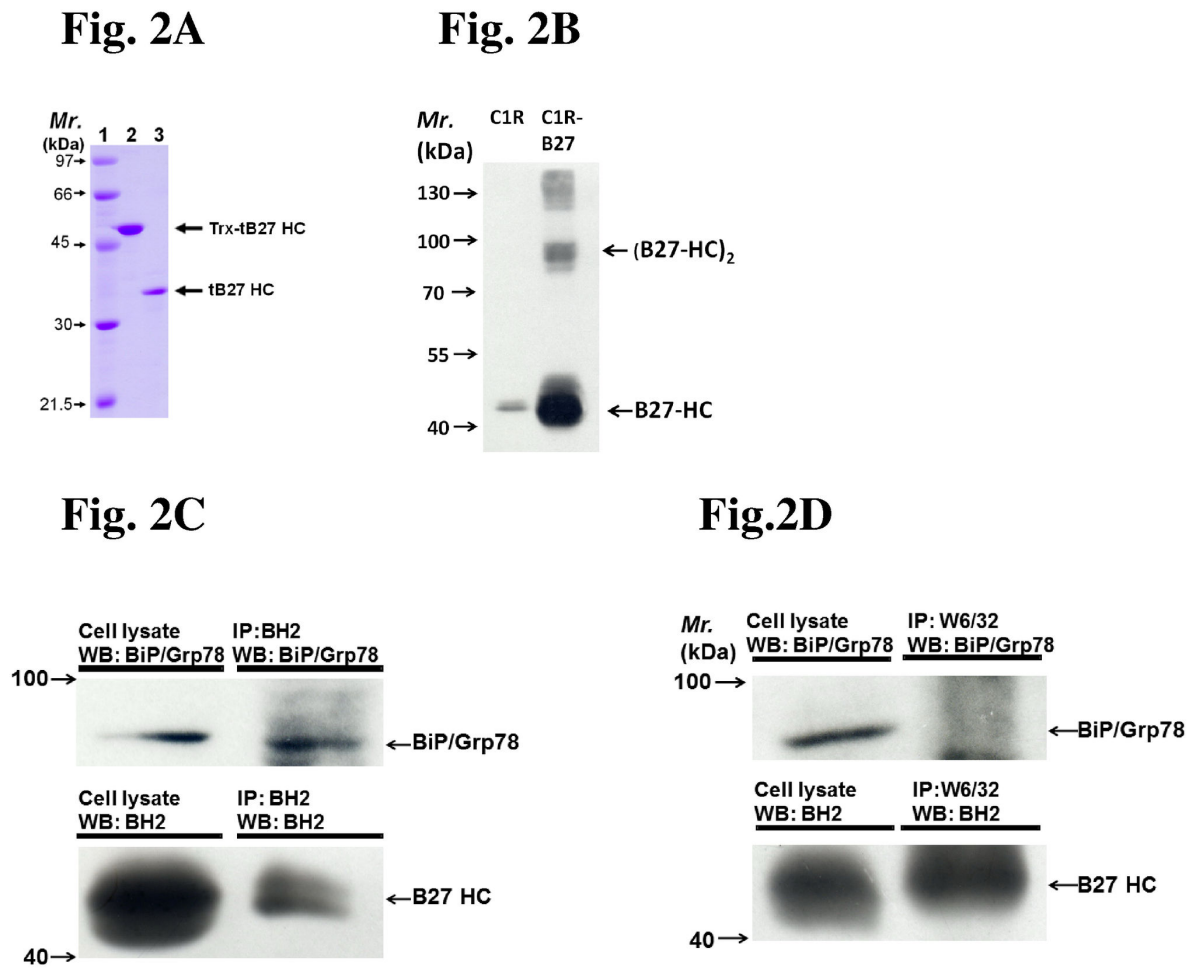
The BH2 monoclonal antibody recognizes the misfolded HLA-B27 HC (A) Trx-truncated HLA-B27 HC and truncated HLA-B27 HC were resolved by SDS-PAGE (12%) and stained with Coomassie Brilliant Blue. Lane 1: Molecular weight markers. Lane 2: 2 μg of Trx-truncated HLA-B27 HC. Lane 3: 2 μg of truncated HLA-B27 HC. (B) BH2 recognizes the monomeric and oligomeric forms of HLA-B27 HC. The membrane proteins from C1R-B2704 cells were extracted, resolved by non-reducing SDS-PAGE (10%), and analyzed by western blot using the BH2 monoclonal antibody (1:1000 dilution). Lane 1: 50 μg of membrane protein extracted from HMy2.C1R cells. Lane 2: 50 μg of membrane protein extracted from C1R-B2704 cells. (C) The BiP (Grp 78)/misfolded HLA-B27 HC complex can be immunoprecipitated by BH2. Cell lysate (50 μg) and the immunoprecipitated product from cell lysate by BH2 were analyzed by western blotting and probed for BiP/Grp 78 or HLA-B27 HC (recognized by BH2). (D) The HLA-B27 immunoprecipitated by W6/32 does not bind to BiP/Grp 78. Cell lysate (50 μg) and the immunoprecipitated products from cell lysate by W6/32 were analyzed by western blotting and probed for BiP/Grp 78 or HLA-B27 HC.

A recombinant truncated HLA-B2704 HC was produced for preparation of anti-misfolded HLA-B27 HC monoclonal antibody (Figure 2A) and monoclonal antibody BH2 was chosen. The membrane proteins of C1R-B2704 cells were extracted, separated by the non-reducing SDS-PAGE, and analyzed by western blot using BH2. BH2 recognized monomeric, dimeric, and oligomeric membrane-bound HLA-B27 HC (Figure 2B). The high molecular weight species of HLA-B27 HC complexes were eliminated by reduction prior to SDS-PAGE analysis, suggesting that their formation was due to self-association by disulfide linkages (Figure S4). Whether HLA-B27 HC recognized by BH2 is the misfolded/unfolded form was also examined. HLA-B27 HC over-expressed in HMy2.C1R cells was extracted and immunoprecipitated by BH2. The misfolded/unfolded HLA-B27 HC has been found to associate with BiP/Grp 78 protein in the ER [1,28]. Figure 2C indicates that BiP/Grp 78 binds to the BH2-precipitated HLA-B27 HC, suggesting that BH2 recognizes the misfolded/unfolded HLA-B27 HC. In addition, the folded HLA-B27 can be immunoprecipitated by W6/32, but does not associate with Bip/Grp 78 (Figure 2D). It is possible that the antibody BH2 can recognize other alleles. The recognition specificity of BH2 needs to be further characterized. 

**Figure 3:**
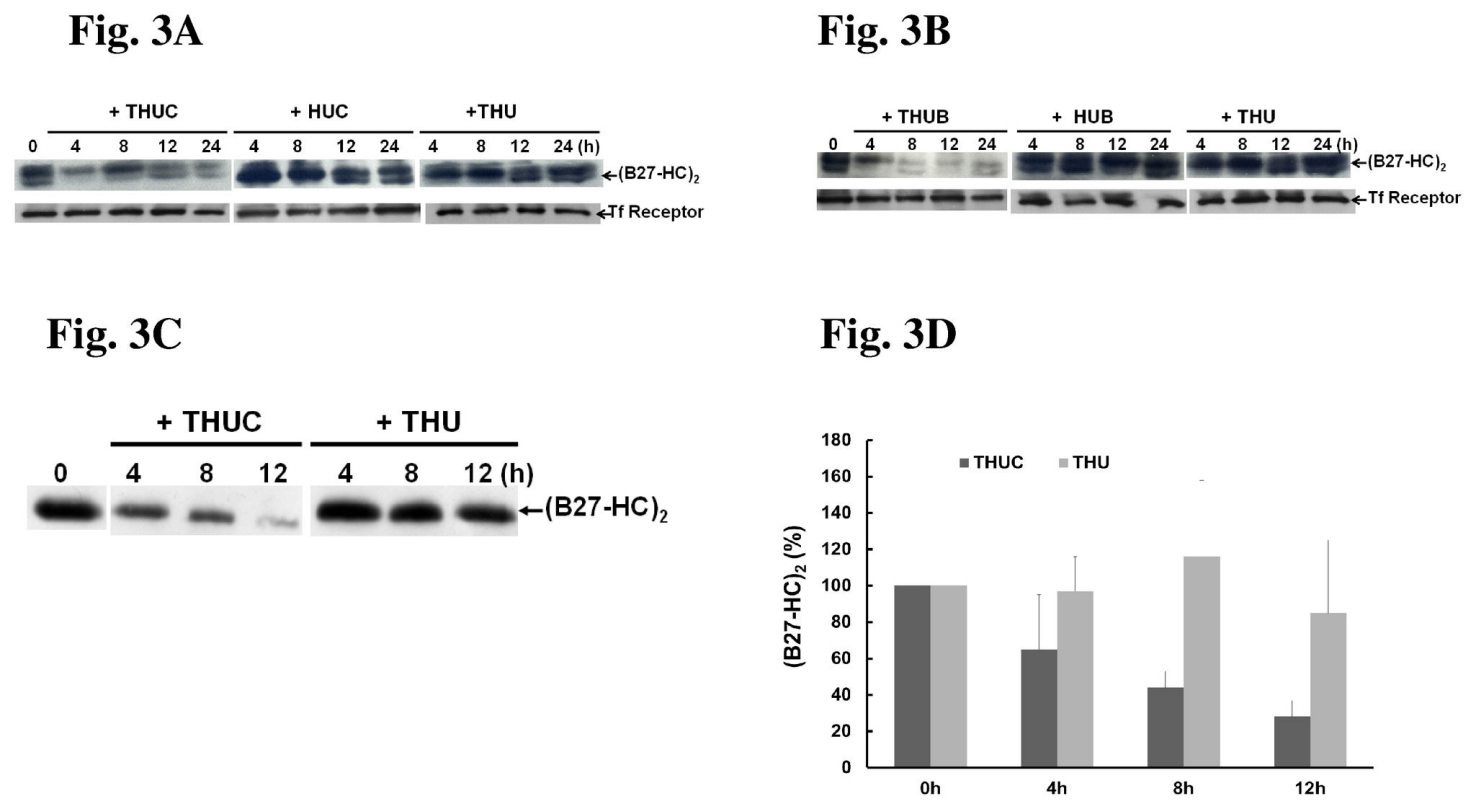
Treatment with either THUC or THUB reduces the formation of (B27-HC)_2_. After treatment with each protein for the indicated time point, membrane proteins were extracted. 50 μg of extract was resolved by non-reducing SDS-PAGE (10%) and immunoblotted with a BH2 monoclonal antibody and anti-transferrin (Tf) receptor antibody. Tf receptor serves as an internal control. (A) THUC treatment for 12 h, but not THU or HUC treatment, significantly decreased the levels of (B27-HC)_2_. (B) The level of (B27-HC)_2_ is significantly reduced in C1R-B2704 cells treated with THUB. (C) The production of (B27-HC)_2_ is reduced when PBMCs isolated from AS patients are treated with THUC. (D) The results obtained in Figure 3C are plotted. The amount of immunostaining observed at 0 h was set to 100%. The results shown are the mean levels of (B27-HC)_2_ immunostaining observed in membrane proteins independently extracted from the PBMCs of five AS patients (mean ± SD, n = 5).

We next examined whether treatment with THUC suppressed the formation of (B27-HC)_2_ in C1R-B2704 cells. The results showed that the level of (B27-HC)_2_ in C1R-B2704 cells was indeed reduced in THUC-treated cells, whereas neither THU nor HUC altered the level of (B27-HC)_2_ (Figure 3A). Treatment of C1R-B2704 cells with THU, HUC, HUB, THUC or THUB does not affect the expression of HLA-B27 HC (Figure S5). In addition, the levels of (B27-HC)_2_ maintain constant over the incubation time in C1R-B2704 cells (Figure S6). Thus, it appears that THUC enters the cells, the cargo peptide is released in the cytosol, and then translocates to the lumen of the ER to promote HLA-B27 folding. Another cargo peptide-bearing protein, THUB, also exerted similar effects in suppression of (B27-HC)_2_ formation (Figure 3B). Neither HUB nor THU was capable of suppressing the production of (B27-HC)_2_ (Figure 3B). In addition to the cell culture model, THUC treatment also reduced the levels of (B27-HC)_2_ in PBMCs isolated from AS patients (Figure 3C and 3D), suggesting that similar results can be achieved in normal differentiated cells. 

**Figure 4:**
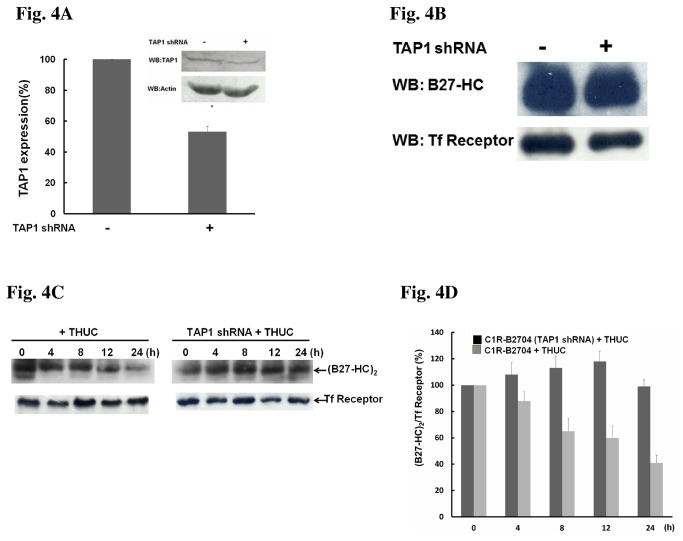
THUC-treatment reducing the level of (B27-HC)_2_ in C1R-B2704 cells is TAP1-dependent. (A) Stably expressed TAP1 shRNA reduces TAP1 expression. (B) Knockdown of TAP1 does not affect the expression of HLA-B27 HC. (C) TAP1-knockdown suppresses the THUC-induced reduction of (B27-HC)_2_. (D)The ratio of (B27-HC)_2_/Tf receptor averaged from three independent experiments in Figure 4C are plotted against THUC treatment time. The ratio of (B27-HC)_2_/Tf receptor extracted from cells without treatment with THUC was set to 100%.

The TAP1/TAP2 complex is a peptide transporter on the ER membrane [29,30], and knockdown of TAP1 expression by shRNA will impair the delivery of antigenic peptides from the cytosol to the ER lumen. As expected, TAP1 expression in C1R-B2704 cells was reduced by shRNA (Figure 4A). Knockdown of TAP1 does not affect the expression of HLA-B27 HC in C1R-B2704 cells (Figure 4B), but resulted in a reduction of THUC-mediated suppression of (B27-HC)_2_ formation (Figure 4C and 4D).

**Figure 5:**
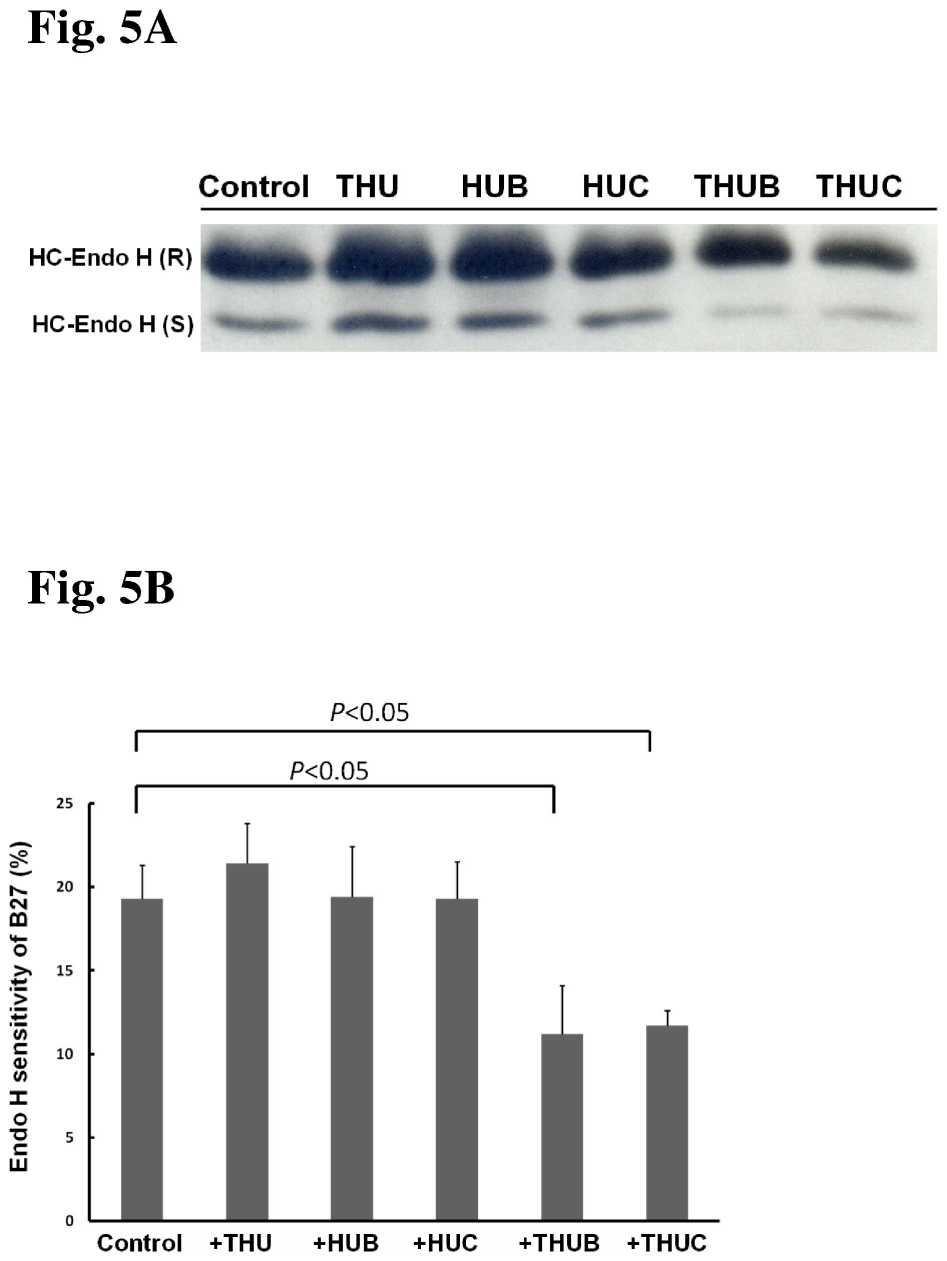
Analysis of HLA-B27 HC by Endo H digestion. (A) SDS-PAGE analysis of Endo H-resistant and Endo H-sensitive HLA-B27 HC in C1R-B2704 cells. C1R-B2704 cells were treated with the indicated reagents. Membrane proteins (2 μg) digested with Endo H (one unit) were analyzed by reducing SDS-PAGE and immunoblotted by BH2 monoclonal antibody. (B) The results obtained in Figure 5A were plotted. The ratio of Endo H-sensitive HLA-B27 HC/total HLA-B27 HC is averaged from three independent experiments (mean ± SD, n =3).

The N-linked glycoproteins in the ER are sensitive to Endo H digestion. When proteins in the ER have been delivered into the Golgi apparatus where the glycan was further modified, they resist to Endo H digestion. The unique specificity of Endo H was used to monitor HLA-B27 HC trafficking [31].Treatment of C1R-B2704 cells with THU, HUB or HUC does not alter the Endo H-sensitive fractions of HLA-B27 HC (Figure 5A and 5B). However, the Endo H-sensitive fractions of HLA-B27 HC were reduced when cells were treated with THUC or THUB (Figure 5A and 5B), suggesting that more folded HLA-B27 molecules have entered the Golgi body. 

**Figure 6:**
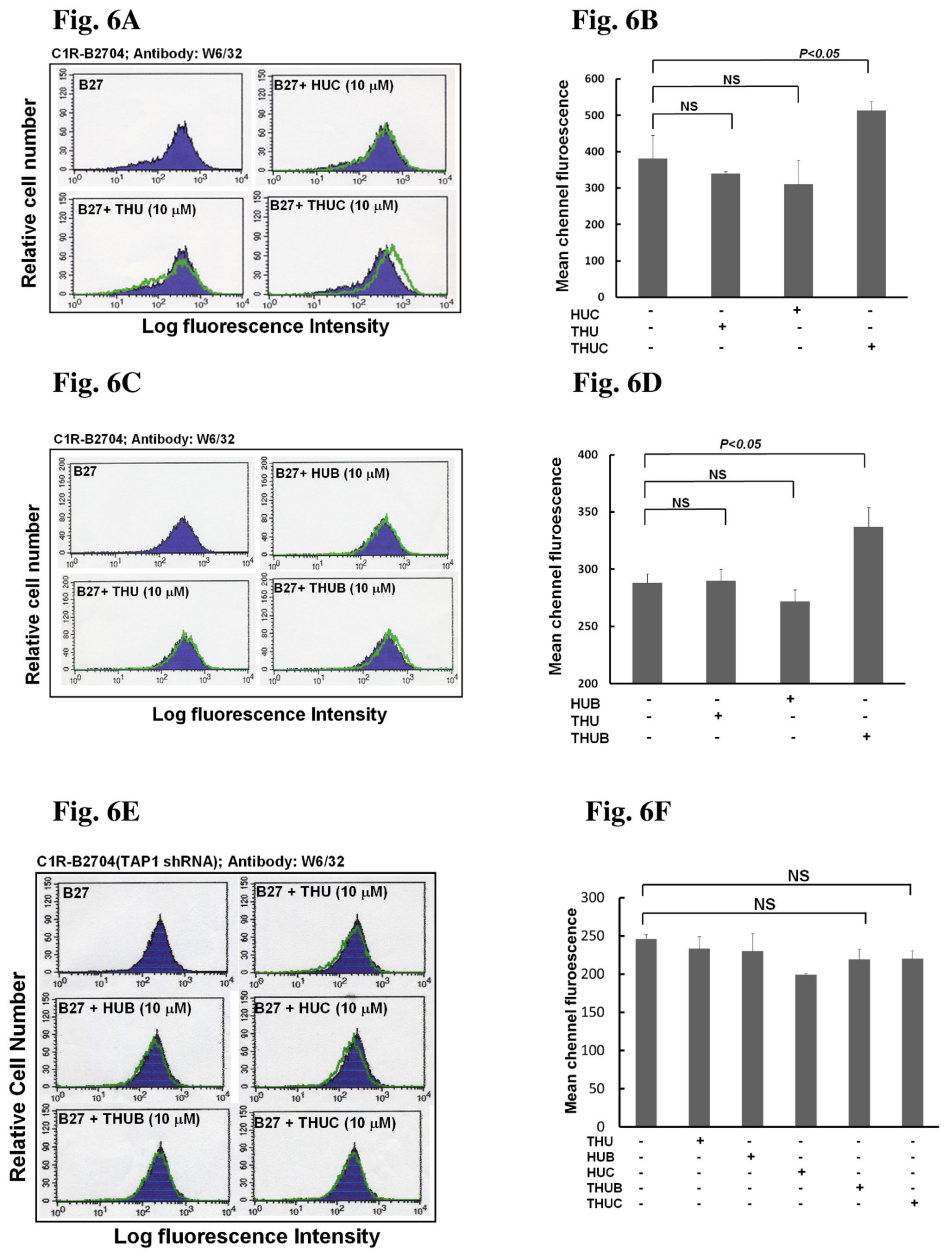
Treatment with either THUC or THUB increases the antigenic peptide targeted to the cell surface of C1R-B2704. (A) Treatment with THUC, but not THU or HUC, increases the HLA-B27 HC/β_2_m/RRFKEGGRGGKY complex presented at the cell surface of C1R-B2704, as detected by flow cytometry. (B) Mean-channel fluorescence measured in Figure 6A is increased when C1R-B2704 cells were treated with THUC. Values (mean ± SD, n=3) are averaged from three independent experiments. (C) Treatment with THUB, but not THU or HUB, increases the HLA-B27 HC/β_2_m/ RRYLENGKETL complex presented at cell surface, as detected by flow cytometry. (D) Mean-channel fluorescence measured in Figure 6C is increased when C1R-B2704 cells were treated with THUB. Values (mean ± SD, n=3) are averaged from three independent experiments. (E) Knockdown of TAP1 impairs the antigenic peptides presented on the cell surface. (F) Mean-channel fluorescence measured in Figure 6E is not affected when C1R-B2704 cells with TAP1 knockdown were treated with THUC or THUB. Values (mean ± SD, n=3) are averaged from three independent experiments.

If the HLA-B27-binding peptide has been delivered to the ER lumen, it will promote HLA-B27 HC folding and bind to the HLA-B27 HC/β_2_m complex. As a result, the amount of assembled HLA-B27/β_2_m/peptide complex will be increased and will be translocated to the cell surface. Therefore, we examined whether the ER-targeted HLA-B27-binding peptide was transported to the cell surface by the HLA-B2704 HC/β_2_m complex. If the HLA-B2704 HC/β_2_m/ peptide complex is present on the plasma membrane, it will be reactive to W6/32, a monoclonal antibody that recognizes the folded HC associated with β_2_m [32]. After C1R-B2704 cells were treated with THUC or THUB, there was a clear increase in the population of W6/32-reactive cells (Figure 6A-6D), suggesting that the cargo peptide was presented on the cell surface by the HLA-B2704 HC/β_2_m complex. Consistent with our previous result showing that the control protein had no effect on the suppression of (B27-HC)_2_ formation, treatment with THU, HUC or HUB did not increase the W6/32-reactive cell population (Figure 6A-6D). In addition, knockdown of TAP1 impairs the translocation of cleaved peptide into the ER, in turn affecting the assembly of HLA-B2704/β_2_m/peptide complex and peptide presentation on the cell surface. Thus, an increase of the cargo peptide presented on the cell surface of TAP-knockdown cells was disappeared even though they were treated with THUC or THUB (Figure 6E and 6F). 

**Figure 7:**
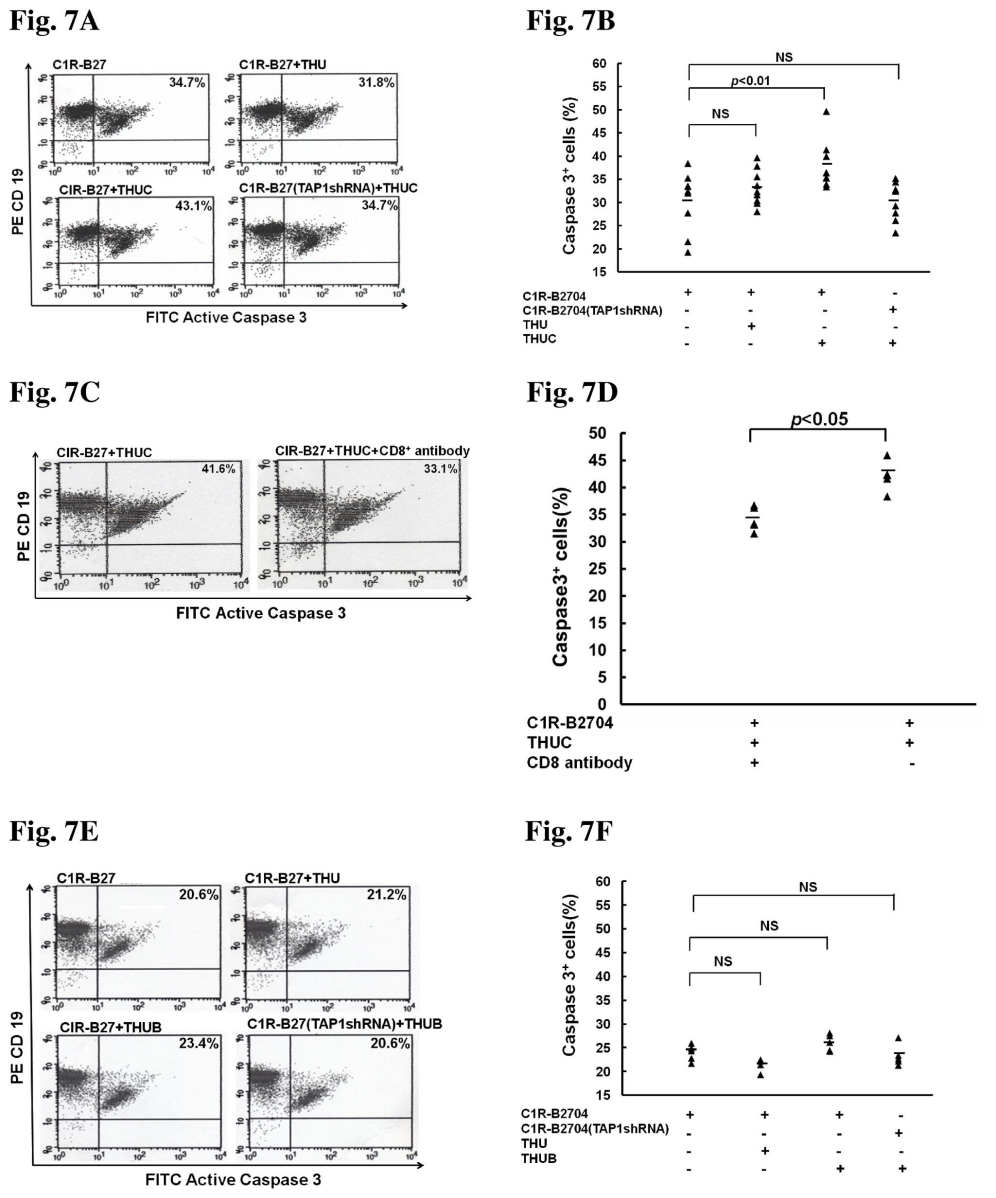
Treatment with THUC, but not THUB or THU, enhances apoptosis mediated by CD8^+^ T-cell cytotoxicity. (A) Treatment with THUC, but not THU, increases apoptosis mediated by CD8^+^ T-cell cytotoxicity. TAP1-knockdown abolishes the THUC-induced increase in apoptosis. PBMCs isolated from AS patients (n = 9) were treated with THUC, and stimulated with IL-2. CD8^+^ T cells were isolated from THUC-stimulated PBMCs. C1R-B2704 cells were stained with phycoerythrin-conjugated anti-CD19 antibodies and the apoptotic cells were stained with FITC-conjugated anti-active caspase 3 antibody. (B) The results obtained in Figure 7A are plotted. (C) The THUC-induced cell apoptosis mediated by CD8^+^ T-cell cytotoxicity is CD8-dependent. (D) The results obtained in Figure 7C are plotted. (E) Treatment with THUB cannot enhance apoptosis mediated by CD8^+^ T-cell cytotoxicity. (F) The resulted obtained in Figure 7E are plotted.

Furthermore, we examined whether the cargo peptide presented by the HLA-B27 HC/β_2_m complex on the cell surface could bind and activate human CD8^+^ T cells. Results of this experiment showed that treatment with THUC, but not THU, increased the apoptosis of C1R-B2704 cells mediated by CD8^+^ T lymphocyte cytotoxicity (Figure 7A and 7B). Sera from eight AS patients have been tested for infection of *Chlamydia trachomatis*. No antibody in the sera against *Chlamydia trachomatis* was found (data not shown). When TAP1 expression was knocked down, treatment with THUC failed to increase cell apoptosis (Figure 7A and 7B), suggesting that the cargo peptide transported into the ER is critical for the cytotoxicity mediated by activated CD8^+^ T cells. We also examined whether THUC increasing cell apoptosis mediated by T lymphocyte cytotoxicity was CD8^+^-dependent. The stimulated T lymphocytes were pre-blocked by anti-CD8 antibody and analyzed by flow cytometry (Figure S7). Pre-treatment of the activated T lymphocytes with anti-CD8 antibody reduced THUC-induced cell apoptosis mediated by activated CD8^+^ T cells (Figure 7C and 7D). Interestingly, although the HLA-B27 HC peptide (RRYLENGKETL) was present on the cell surface after cells were treated with THUB, its presence did not increase the apoptosis of C1R-B2704 cells mediated by CD8^+^ T lymphocyte cytotoxicity (Figure 7E and 7F). 

## Discussion

 We have demonstrated that THU is a useful vehicle to deliver the HLA-B27-binding peptide to the lumen of the ER. HLA-B27-binding peptide present in the ER lumen promotes HLA-B27 HC folding, as evidenced by an increase of W6/32-reactive HLA-B27 on the cell surface of C1R-B2704 after treatment with THUC (Figure 6A and 6B) or THUB (Figure 6C and 6D). Knockdown of TAP1 impairs the translocation of the antigenic peptide (RRFKEGGRGGKY) cleaved from THUC into the ER. Thus, treatment with THUC cannot increase W6/32-reactive HLA-B27 molecules on the cell surface (Figure 6E and 6F). 

 The pathology of AS mainly affects the entheses, where tendons, ligaments, and capsules are inserted into the bone. Early symptoms of AS include an inflammatory reaction of the spine and some patients develop ankylosis and syndesmophytes with time. Conventional medical treatment is mainly based on non-steroidal anti-inflammatory agents; tumor-necrosis factor blocking agents are employed in most non-steroidal anti-inflammatory agent-resistant patients. However, although anti-TNF therapy can improve much of the inflammation, development of bone erosion or syndesmophyte formation cannot be stopped [4,33]. Thus, there is a need for effective treatments to arrest bone erosion and syndesmophyte formation in AS. The pathogenesis of AS possibly arises from the intrinsic propensity of HLA-B27 HC to fold at a slow rate, resulting in accumulation of misfolded HLA-B27 HC in the ER, triggering the UPR, and forming the heavy chain homodimer, (B27-HC)_2_. It is apparent that induction of proper HLA-B27 HC folding is a potential avenue for drug development in AS therapy. In this study, we have developed a novel approach for the delivery of HLA-B27-binding peptides into the ER that can then promote folding of the HLA-B27 HC, reducing heavy chain homodimer formation. 

 We have demonstrated that two HLA-B27-binding peptides, RRFKEGGRGGKY and RRYLENGKETL, can be delivered into the ER by our methods. In the ER, these peptides promote HLA-B27 HC folding, reduce the formation of the heavy chain homodimer (Figure 3A-3D), form a complex with HLA-B27 HC and β_2_m, and are translocated to the cell surface (Figure 6A and 6C). It will be interesting to determine whether the enhanced HLA-B27 HC folding due to the delivered HLA-B27-binding peptide can release the inflammatory and arrest bone erosion in an AS animal model; more basic studies in an AS animal model using the peptide therapy will be required in the future. 

 Although our delivered peptides can be presented on the cell surface by HLA-B27 molecules, only the peptide derived from the DNA primase of *Chlamydia trachomatis* induced cytotoxicity mediated by the T-cell receptors of CD8^+^ T lymphocytes (Figure 7A and 7B). The other peptide tested, derived from the human HLA-B27 HC, failed to induce cytotoxicity (Figure 7E and 7F). This result suggests that the host has developed T-cell receptors that specifically recognize the bacterial peptide presented by HLA-B27 molecules. A host is generally tolerant to peptides derived from self-proteins, accounting for the observation that peptide derived from human HLA-B27 HC presented on the cell surface by HLA-B27 molecules failed to generate T-cell receptors mediating cytotoxicity. 

 Antigenic peptides derived from host or foreign proteins are generated in the cytoplasm by proteasomes. The correct length and sequence are critical for the antigenic peptides to be stably assembled with HLA class I molecules. Proteasomes usually generate peptides of 2-25 amino acids in length, but only ^~^15% of these peptides are of the appropriate length for optimal binding to HLA-class I molecules [34,35]. It is known that proteasomes generate the C-terminus of the final antigenic peptide epitope [36] and then the antigenic peptide precursors are transported into the lumen of ER by TAP, where the N-terminal extension of some peptides is tailored by aminopeptidases to the final length [37-39]. Peptide-tailoring is a critical step in determination of the rate of peptide loading, and the folding rate of HLA molecules is governed by the peptide-loading step [40,41]. Indeed, a slow peptide-tailoring rate is associated with the development of AS. Endoplasmic reticulum-associated aminopeptidase 1 (ERAP1) is one of the peptide-tailoring peptidases and has been identified as one of the AS susceptibility genes [42]. Our method described here can deliver the appropriate length and correct sequence of HLA-B27-binding peptide into the ER without additional tailoring by aminopeptidases, accelerating HLA-B27 folding and reducing HLA-B27 HC misfolding. Such peptide therapy is a potential approach for the treatment of AS induced by defective ERAP1. 

 In the transgenic rat model, up-regulation of HLA-B27, resulting in accumulation of misfolded heavy chains, was highly linked to induction of the UPR [15-17]. The signature of UPR, including up-regulation of mRNA for Grp78, CHOP, and XBP-1 transcription factor, was identified in bone marrow macrophages isolated from HLA-B27-transgenic animals [15]. However, recent studies have indicated that pre-treatment with IFNγ to up-regulate HLA-B27 expression in macrophages isolated from AS patients failed to induce the UPR [43]. Up-regulation of the UPR in C1R-B2704 was also not observed in the present study. Thus, whether transport of HLA-B27-binding peptide into the ER can suppress the induction of UPR in macrophages of B27 transgenic rats needs to be determined.

 As demonstrated by these data, we have developed a convenient methodology to deliver an antigenic peptide to the ER for subsequent presentation on the cell surface by MHC class I molecules. This approach has potential applications in the development of peptide therapy not only for AS, but also immunotherapy for cancers. For example, a suitable antigenic peptide could be delivered to tumors using THU, allowing cancer cells to present the delivered peptide on their plasma membrane. Our vehicle for translocation could also deliver the same antigenic peptide to dendritic cells for stimulation and amplification of the CD8^+^ T cells that specifically recognize the presented antigenic peptide. The activated CD8^+^ T cells could be isolated and introduced for killing of cancer cells that present the delivered antigenic peptide on their plasma membrane.

## Supporting Information

Table S1 **Sequences of primers used for PCR.**
(TIF)

Figure S1 **SDS-PAGE analysis of THUC or THUB digested by yeast ubiquitin C-terminal hydrolase 1.**
(TIF)

Figure S2 **Analysis of the yeast ubiquitin C-terminal hydrolase 1-cleaved THUC by MALDI-TOF.**
(TIF)

Figure S3 **Analysis of the peptide targeting by fluorescence microsope.**
(TIF)

Figure S4 **Membrane proteins extracted from C1R-B2704 cells are analyzed by SDS-PAGE (10%).**
(TIF)

Figure S5 **Treatment of C1R-B2704 cells with THU, HUB, HUC, THUB or THUC does not affect the expression levels of B27 HC.**
(TIF)

Figure S6 **The levels of (B27-HC)_2_ maintain constant over the incubation time in C1R-B2704 cells.**
(TIF)

Figure S7 **Analysis of the CD8^+^ T cells pre-blocked with anti-CD8 antibody by flow cytometry.**
(TIF)
