# Supplementary figures and images for: Targeted Delivery of an Antigenic Peptide to the Endoplasmic Reticulum: Application for Development of a Peptide Therapy for Ankylosing Spondylitis
Source: PLoS One. 2013 Oct 14;8(10):e77451. doi: 10.1371/journal.pone.0077451 (PMC3796468; doi:10.1371/journal.pone.0077451)

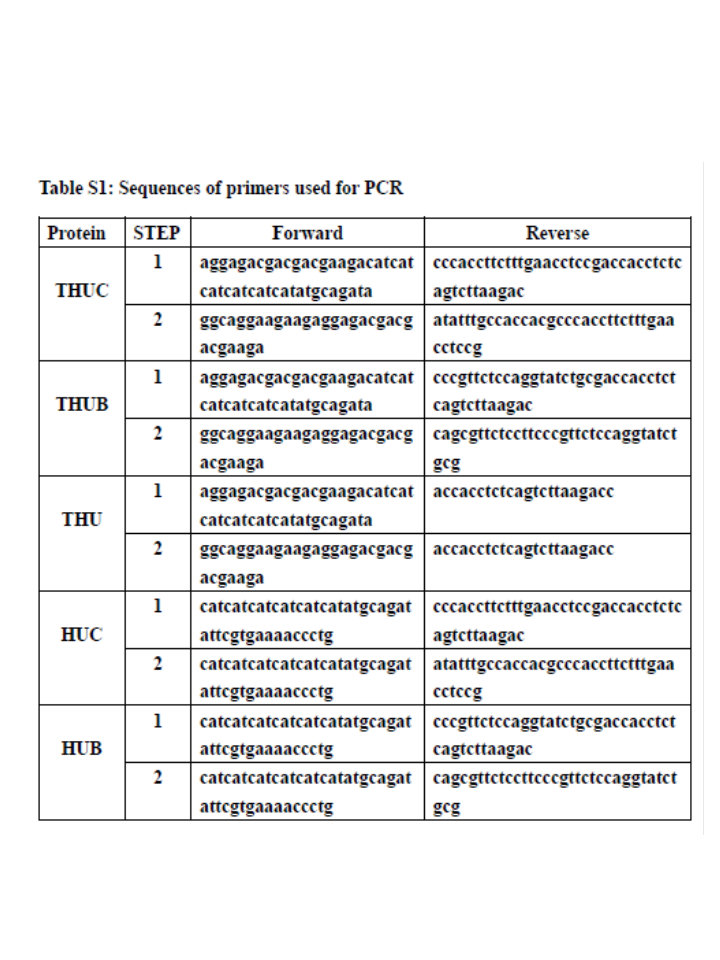

Supplement: Table S1 — Sequences of primers used for PCR. (TIF) [file pone.0077451.s001.tif]

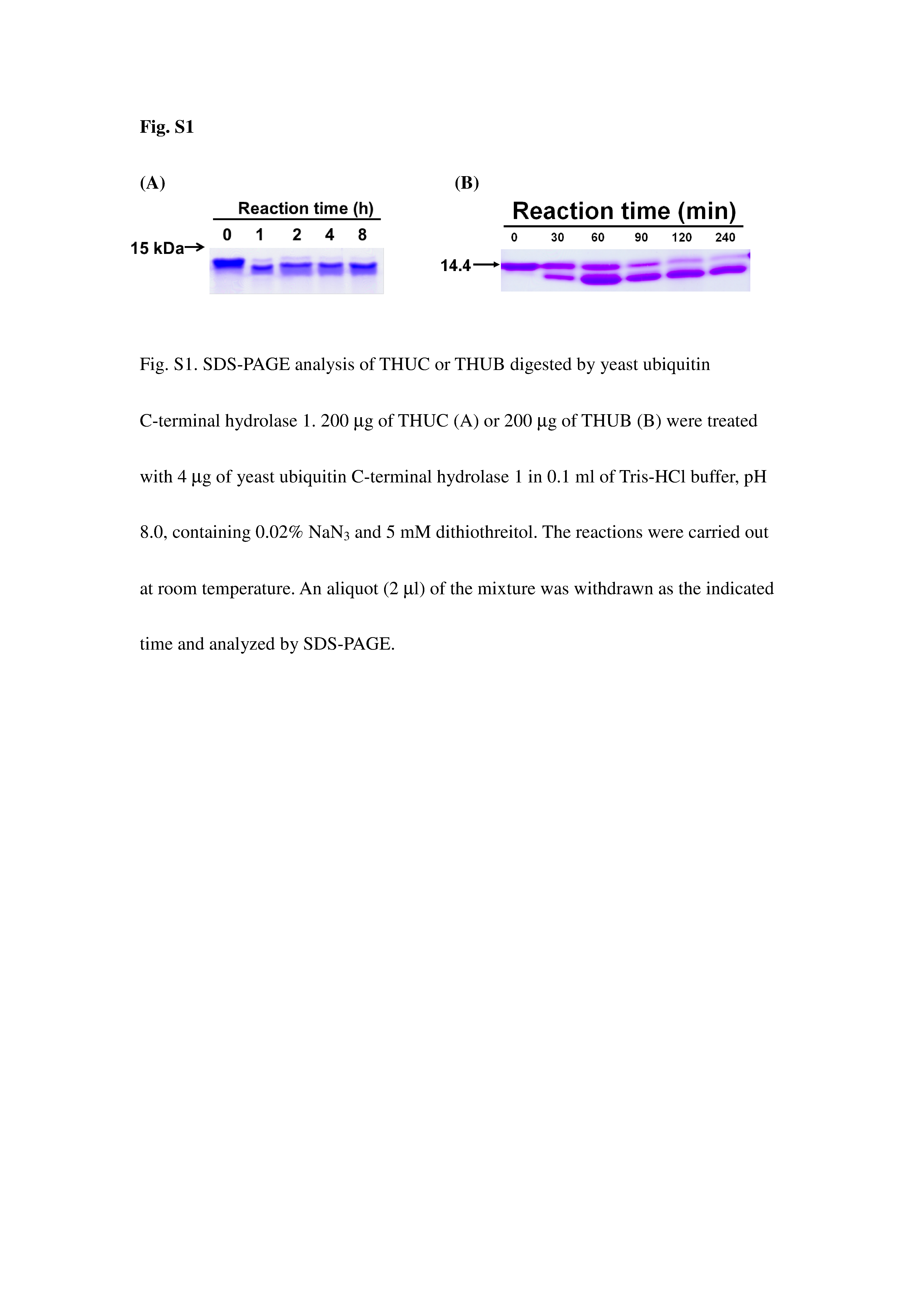

Supplement: Figure S1 — SDS-PAGE analysis of THUC or THUB digested by yeast ubiquitin C-terminal hydrolase 1. (TIF) [file pone.0077451.s002.tif]

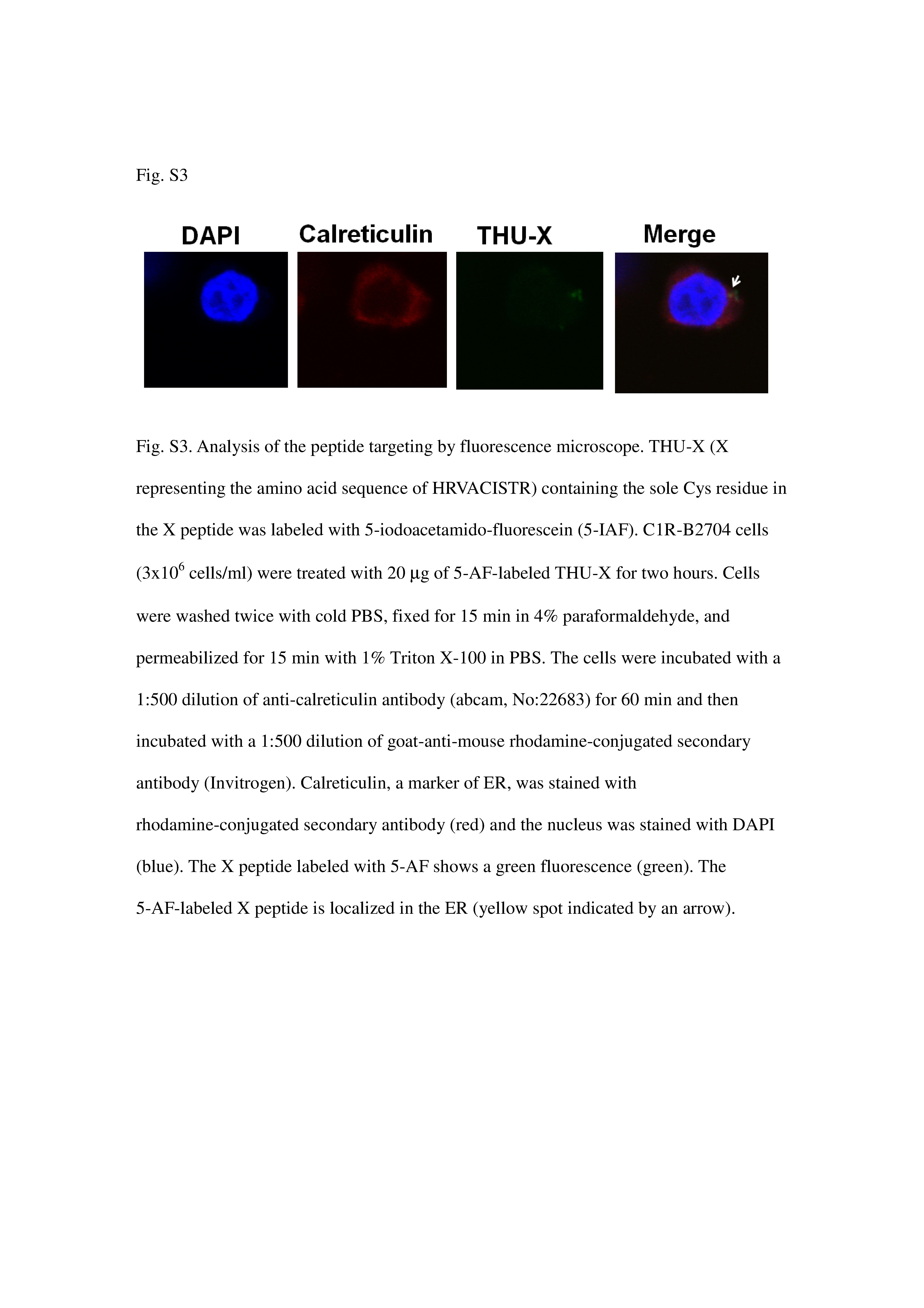

Supplement: Figure S3 — Analysis of the peptide targeting by fluorescence microsope. (TIF) [file pone.0077451.s004.tif]

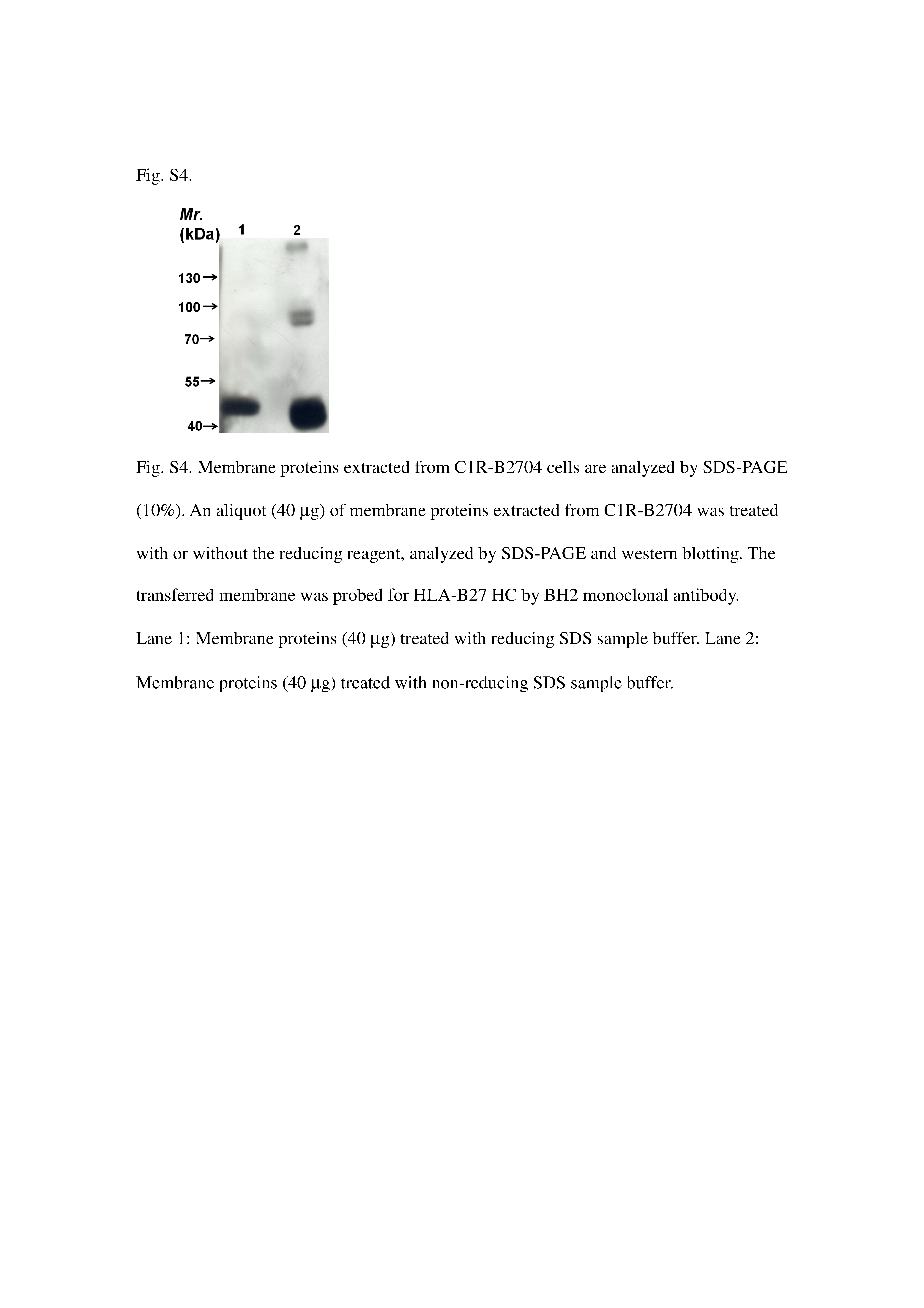

Supplement: Figure S4 — Membrane proteins extracted from C1R-B2704 cells are analyzed by SDS-PAGE (10%). (TIF) [file pone.0077451.s005.tif]

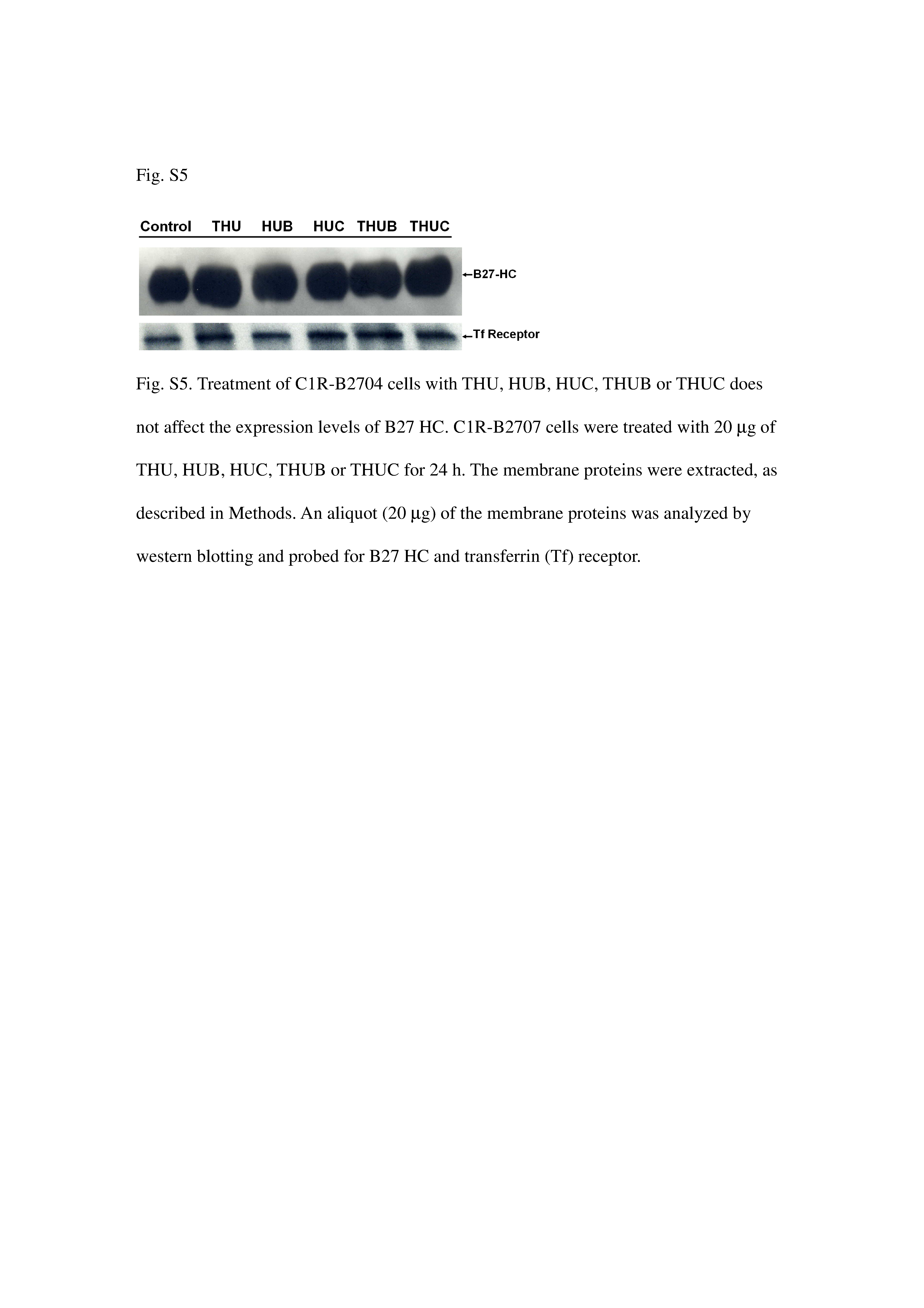

Supplement: Figure S5 — Treatment of C1R-B2704 cells with THU, HUB, HUC, THUB or THUC does not affect the expression levels of B27 HC. (TIF) [file pone.0077451.s006.tif]

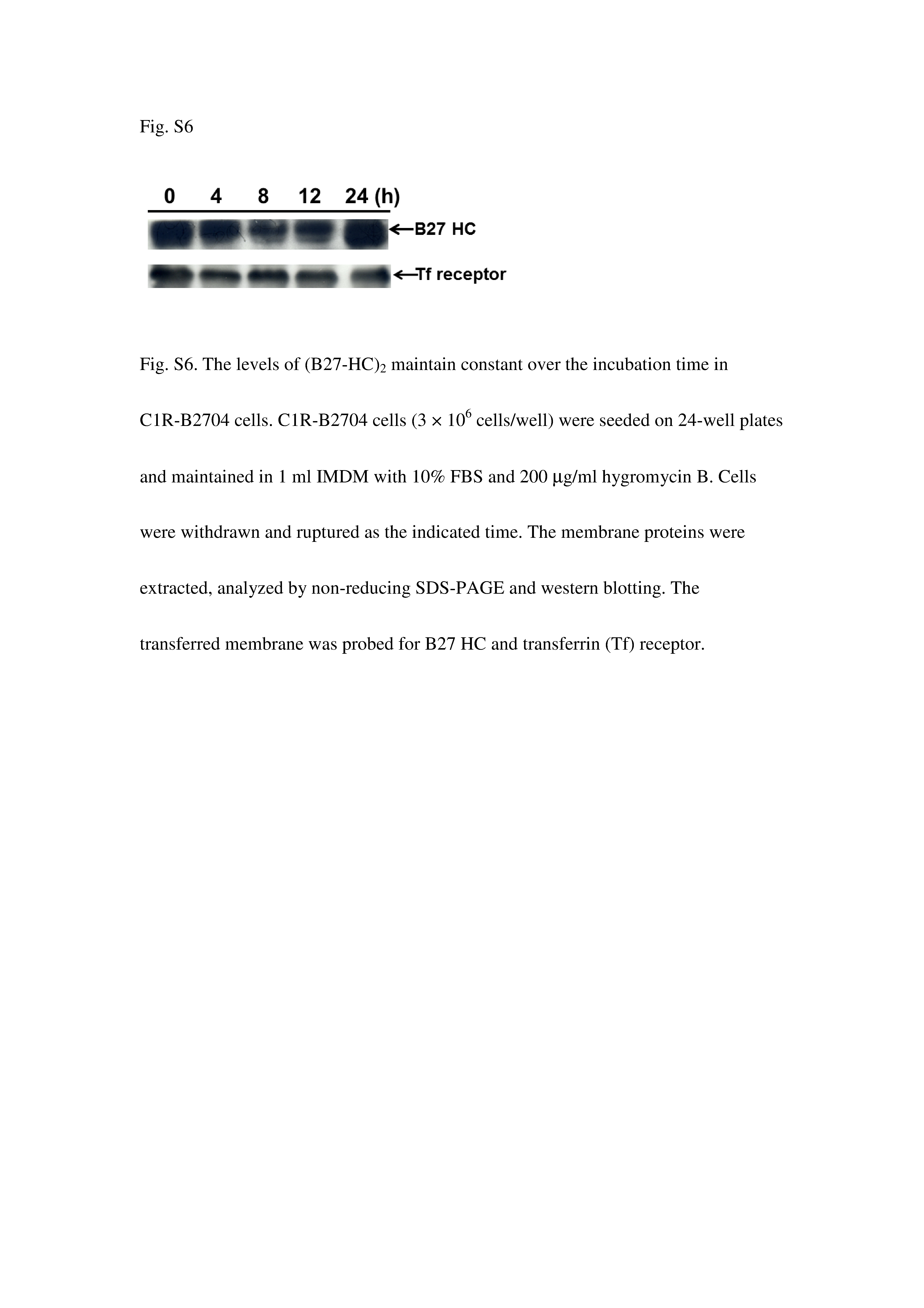

Supplement: Figure S6 — The levels of (B27-HC)2 maintain constant over the incubation time in C1R-B2704 cells. (TIF) [file pone.0077451.s007.tif]

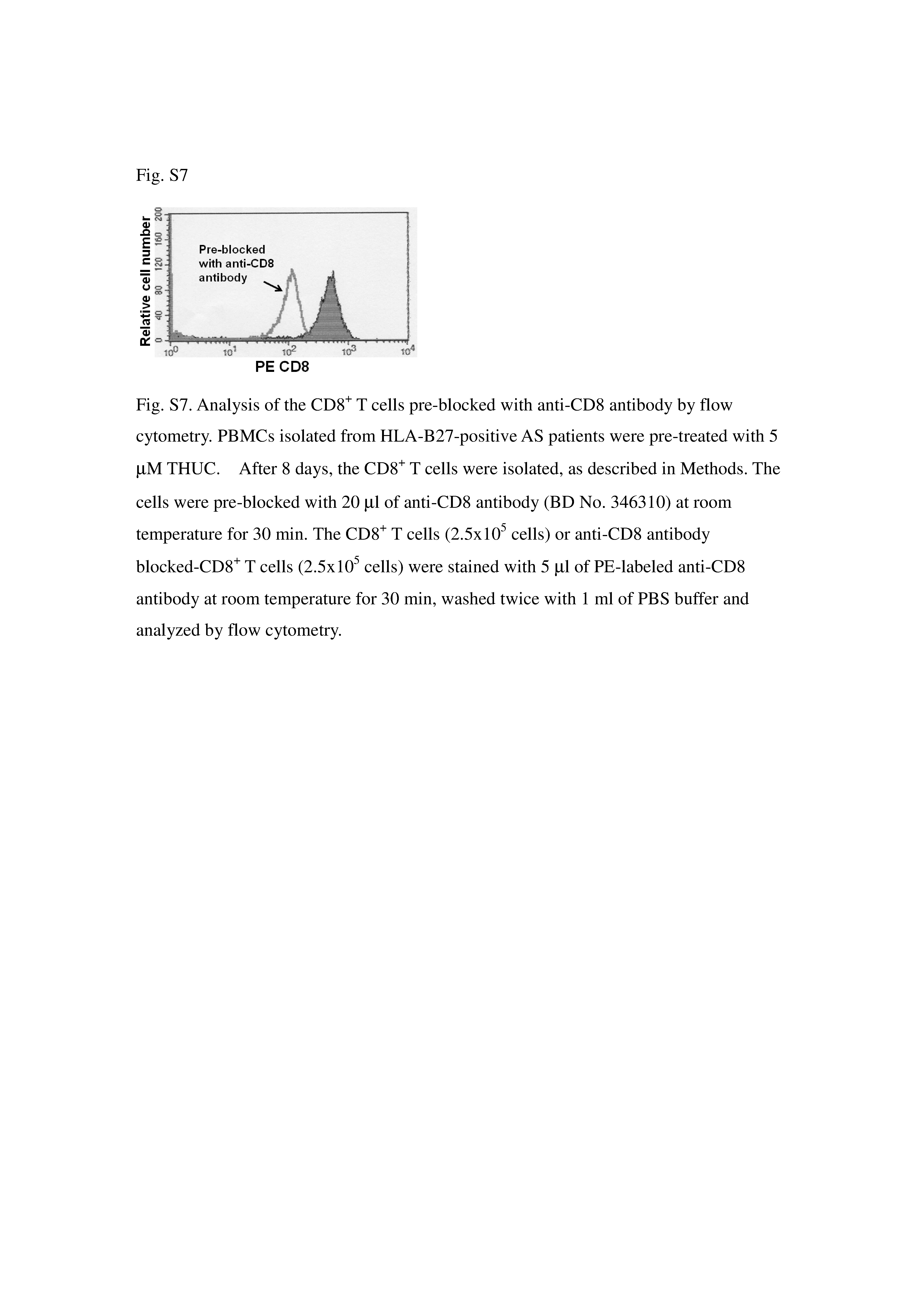

Supplement: Figure S7 — Analysis of the CD8+ T cells pre-blocked with anti-CD8 antibody by flow cytometry. (TIF) [file pone.0077451.s008.tif]
